# A Flexible Multimodal Sole Sensor for Legged Robot Sensing Complex Ground Information during Locomotion

**DOI:** 10.3390/s21165359

**Published:** 2021-08-09

**Authors:** Yingtian Xu, Ziya Wang, Wanjun Hao, Wenyu Zhao, Waner Lin, Bingchen Jin, Ning Ding

**Affiliations:** 1 Shenzhen Institute of Artificial Intelligence and Robotics for Society (AIRS), Shenzhen 518129, China; xuyingtian@cuhk.edu.cn (Y.X.); wanjunhao@cuhk.edu.cn (W.H.); zhaowenyu@cuhk.edu.cn (W.Z.); linwaner@sjtu.edu.cn (W.L.); bingchenjin@cuhk.edu.cn (B.J.); dingning@cuhk.edu.cn (N.D.); 2 Institute of Robotics and Intelligent Manufacturing, The Chinese University of Hong Kong, Shenzhen 518172, China

**Keywords:** flexible sensor, multimodal haptic perception, legged robot

## Abstract

Recent achievements in the field of computer vision, reinforcement learning, and locomotion control have largely extended legged robots’ maneuverability in complex natural environments. However, little research focuses on sensing and analyzing the physical properties of the ground, which is crucial to robots’ locomotion during their interaction with highly irregular profiles, deformable terrains, and slippery surfaces. A biomimetic, flexible, multimodal sole sensor (FMSS) designed for legged robots to identify the ontological status and ground information, such as reaction force mapping, contact situation, terrain, and texture information, to achieve agile maneuvers was innovatively presented in this paper. The FMSS is flexible and large-loaded (20 Pa–800 kPa), designed by integrating a triboelectric sensing coat, embedded piezoelectric sensor, and piezoresistive sensor array. To evaluate the effectiveness and adaptability in different environments, the multimodal sensor was mounted on one of the quadruped robot’s feet and one of the human feet then traversed through different environments in real-world tests. The experiment’s results demonstrated that the FMSS could recognize terrain, texture, hardness, and contact conditions during locomotion effectively and retrain its sensitivity (0.66 kPa^−1^), robustness, and compliance. The presented work indicates the FMSS’s potential to extend the feasibility and dexterity of tactile perception for state estimation and complex scenario detection.

## 1. Introduction

Terrain condition is one of the most concerning problems for mobile robots, researchers have tried all kinds of designs and methods to overcome obstacles. Wheeled robots with suspension systems and robots with crawlers are the most common designs to improve off-road ability. Some robots use a hybrid aerial/terrestrial robot system to fly over obstacles [1]. Legged robots are another promising bionic design in all-terrain missions. Over past decades, legged robots have achieved running on the ground, climbing upstairs, and obstacle avoidance, showing their prospects to traverse complex ground conditions by adopting delicate control algorithms and extensive sensors, such as cameras, light detection and ranging (LiDAR), inertial measurement unit (IMU), force/torque (F/T) sensors, and robotic skin. However, it is still barely able to compete with humans or animals. One great gap is that humans and animals have richer tactile perceptions of ontology status and external environments than robots.

Vision sensing as an exteroceptive perception is widely used in legged robots. It can only detect the surface condition and lacks the sensing of physical properties; thus, it is ideal for navigation and planning before interactions happen. However, during the foot–ground interaction, studies have shown that humans and animals heavily depend on tactile sensing, which gives a complete picture of the foot–ground interaction [2]. With rich sensing, the robotic system should be able to blindly generate the basic pattern of robustly stable dynamic locomotion [3]. In other words, the higher level of postural equilibrium depends on a complex fusion of vestibular, visual, proprioceptive, and exteroceptive receptors [4].

To achieve agile maneuvers, legged robots take advantage of legged locomotion, which only consists of point contact with the ground, and the leg is moved through the air. However, they struggle to keep dynamic stability in harsh environments and suffer from the slip phenomenon, which is common and highly difficult to model [5]. Tactile perception, an important part of exteroceptive sensing, has an advantage over visual and space locomotion data, especially in scenarios, such as where the physical properties of the terrain are unknown (unstructured environment), which may arouse crumbling when traversing loose ground or slipping when walking on a slippery surface.

Many early legged robots use F/T sensors to track ground reaction forces (GRFs) and torque, such as ASIMO [6], KHR-3 [7], iCub [8], LOLA [9], and WABIAN-2 [10]. The F/T sensors are used in a series in robots’ wrists to achieve feedback control and gait analysis. Combining the information of specific orientations of strain gauges, the 6-axis F/T sensors can provide a full picture of all directional forces, but at the cost of high impact and inertia in the limbs due to their weight. While robots such as NAO [11] and Walker (UBTECH Robotics, Inc.) integrate piezoresistive sensor arrays in their big feet to detect not only GRFs but also GRFs’ distribution; the characteristic thinness and lightweight of piezoresistive force-sensing resistors (FSR) make it possible to put the sensors into joints or soles without changing the leg design [12]. Arrays of the force sensor not only help robots calculate their center of mass (COM) or zero-moment point (ZMP) but also provides the ability to detect limited terrain and contact information. ANYmal uses series elastic actuators (SEAs) with precise torque sensing in all joints [13]. F. Jenelten et al. have made ANYmal walk blindly over ice through sensor fusion and slippage estimator [4]. Slip is one of the key challenges for legged robots, but the slip algorithm and estimator used now are complex and inaccurate because robots cannot directly sense the slip on their feet.

In recent years, new applications demanded new features, such as mechanical flexibility and conformability, and accordingly, new designs and materials for robotic tactile sensing. Diversified flexible sensors, such as a tactile sensor array [14], three-axis force sensor [15], slip detection sensor [16], and texture surface recognition sensor [17], can help robots get environmental information. While the development of tactile sensors for robotic fingertips and hands continued, the application areas, such as motion planning in an unstructured environment, brought whole-body sensing to the fore [18]. In this study, we try to integrate rich tactile information, such as slippage, contact state, texture, and hardness, into a multimodal sensor for giving robots the ability to directly haptic sensing. G. Cheng et al. reported a multimodal sensor integrating proximity, normal force, acceleration, and temperature for humanoid robots [19]. However, in-plane integration of different sensors leads to heterogeneity at different points and cuts off the connection between multimodal messages. Multilayered sensor arrays in a 3D lattice allow greater integration and synchronicity of different sensor modalities that are better suited for the robot sole. Z. Huang et al. reported a stretchable human–machine interface with a four-layer design that offered multimodal sensing [20]. H. Chen et al. developed an electronic skin based on the piezoelectric and triboelectric effects that can sense minuscule stimuli and respond very fast [21]. Overall, the idea of a multimodal sensor is appealing and novel due to the inherent importance of contact sensing for legged robot control, estimation algorithms, contact switch detection, and so on. It is challenging to apply these intricate electronic skin devices into a complex natural environment, especially as a sole sensor. The fabrication and integration of electronic skin with a high spatiotemporal resolution that is lightweight provides tremendous potential applications.

**Figure 1:**
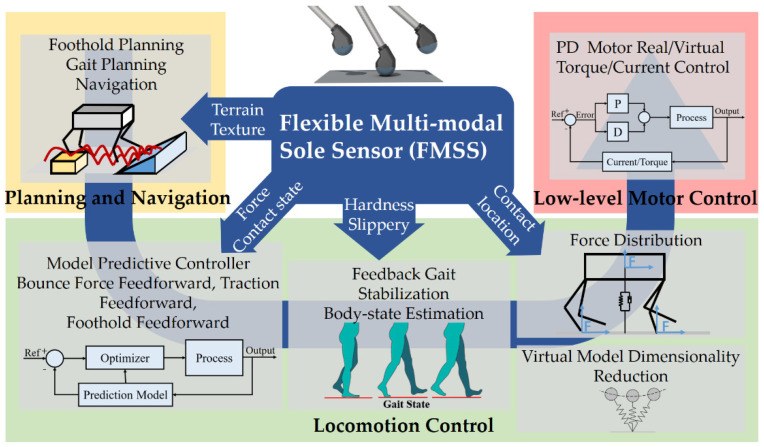
Potential application of FMSS in the whole agile legged robot dynamic control loop.

As tactile sensors can offer key abilities, the tactile force sensor array can be integrated into the hierarchical whole-body controller for tracking contact force and ZMP to provide more stable and agile feedback. The shear force and friction sensing can detect slippage more directly compared to leg-acceleration and velocity-based slip detectors. The texture and hardness sensing can quickly recognize the contact condition and environment. Furthermore, to meet the demand of directly and closely contacting the ground, taking advantage of precise, synchronous sensing, tactile sensors must be compliant, light, robust, and easily integrated into the foot. We proposed a biomimetic flexible multimodal sole sensor (FMSS) design with a large loading capacity for legged robots, which can detect tactile data, such as texture, terrain, force distribution, contact state, and hardness. Richer tactile information applied to hierarchical control can help improve the legged robot into a higher level of sensing fusion and bionic capability from aspects of planning and locomotion control to the low-level control aspect, as seen in Figure 1.

## 2. Materials and Methods

### 2.1. Device Design and Fabrication

Different from other multimodal tactile sensors for legged robots, which usually require a redesign of the whole foot, a soft but durable design for an easy installation is required. Sensors detecting temperature, moisture, proximity, and acceleration are easy to integrate into a multimodal sensor [22], but some of them present little relation to the foot–ground contact process, and some are redundant with the function of robots’ cameras or IMUs. The design picks the most concerning features, such as force, contact state, texture, and hardness, to integrate into a practical multimodal sensor. The sensor weighs about 8 g overall, having a quite small inertial addition for robots. The raw signals are stable enough, uncoupled, and have a high frequency of about 1000 Hz, which reduces the design difficulty and complexity of the acquisition circuit and the whole system [18]. The outermost ground-contact material is ethylene-vinyl acetate copolymer (EVA), a common insole material used in shoes, showing good durability and cushioning.

**Figure 2:**
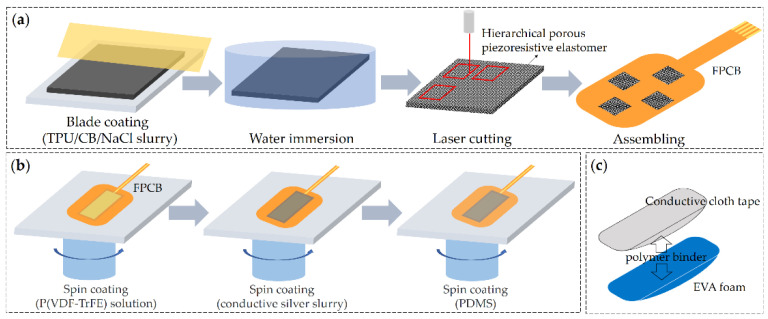
Fabrication of the individual devices, including: (**a**) piezoresistive sensor array, (**b**) piezoelectric sensor, and (**c**) triboelectric sensor.

Considering normal FSRs have a limited measurement range, the special piezoresistive sensor array should cover a large range for measuring the impact force during movement. The finely powdered NaCl particles were employed as the sacrificial template to simultaneously enhance the sensing range and sensitivity of the piezoresistive device [23]. A certain ratio of NaCl (sizes: 50–100 µm), carbon black (CB, TIMCAL), and thermoplastic polyurethanes (TPU), which was dissolved in N, N-Dimethylformamide (DMF) solvent at a 1:3 weight ratio, were mixed thoroughly using a planetary vacuum mixer (HM800, HASAI) to obtain the coating slurry. As shown in Figure 2a, the slurry was then prepared as a 2.0 mm film by blade coating. After curing at 80 °C for 4 h, the film was immersed in water for 24 h, where the water was refreshed every 4 h. Finally, the piezoresistive film was dried at 80 °C for 2 h. For the piezoelectric sensor, the P(VDF-TrFE) (Piezotech)/DMF solution was spin-coated on the flexible printed circuit board (FPCB) with a designed electrode pattern and annealed at 140 °C for 2 h in an oven. Then the conductive sliver slurry (Coldstones Tech) and the encapsulation layer of PDMS were coated by spin casting in turn, followed by baking at 60 °C for 4 h, respectively (Figure 2b).

**Figure 3:**
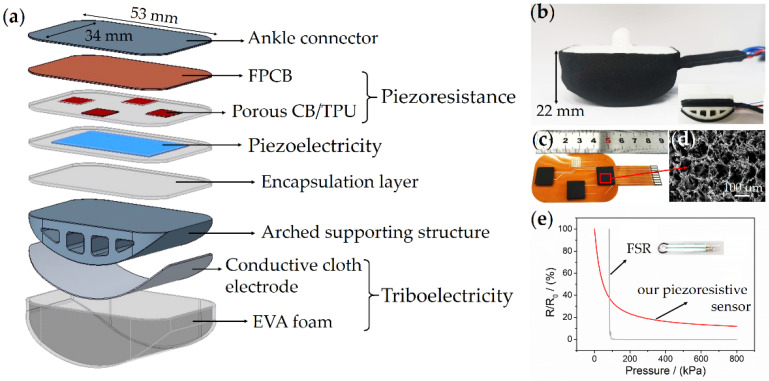
Design and components of the FMSS. (**a**) A cross-section depiction and structural schematic of the FMSS. (**b**) Photographs of the assembled FMSS integrated into the foot structure of the quadruped robot: Pegasus. (**c**) Photographs of the assembled piezoresistive sensor array. (**d**) SEM image showing the sponge-like hierarchical porous piezoresistive elastomer. (**e**) The relative change in resistance (R/R_0_) of the piezoresistive sensor and the FSR, as a function of pressure (0–800 kPa).

The overall design of FMSS is illustrated in Figure 3a, which consists of three synergistic sensing components and sizes, overall 53 × 34 × 22 mm, which refers to the original foot sole, avoiding the problems of readjusting the control code and recalibration of the foot. The shell layer structure is a wear-resistant EVA foam assembled with a flexible conductive cloth electrode for the identification of induced electrical signals from charged objects. This single electrode triboelectric sensor was fastened to the rigid arched supporting structure by a polymer binder (Figure 2c). The triboelectricity layer has to contact the measured object directly to distinguish the texture, so the triboelectricity layer is conformally wrapped on the outermost layer. Because piezoelectricity responds to impulsive deformation, the piezoelectric sensor, which was encapsulated in the two pieces of EVA foam films, was fixed on top of the arched supporting structure. Layers in piezoresistance need flat full-face contact and a hard substrate to map the force distribution, so they are put under the hard ankle connector and above the the piezoelectric sensor. The cross-arranged piezoresistive array was assembled by four laser-cut hierarchical porous piezoresistive cells (sizes of about 10 × 10 mm) and an FPCB with interdigital electrodes (Figure 2a and Figure 3c) [24,25]. The cross-sectional image of a field-emission scanning electron microscope (FESEM, Supra 55 Sapphire, ZEISS) shows abundant micro- and nano-pores distributed into the conductive elastomer (TPU) uniformly (Figure 3d). Plenty of CB nanoparticles are found across the surface of pore walls resulting in a large number of intricate conductive networks, helping with improving device sensitivity. The integrated piezoresistive sensor was fixed on the lower plane of the ankle connector while also placed on the piezoelectric sensor. Figure 3e shows the typical resistance response curves of the piezoresistive sensor and the commercial FSR, respectively. It can be seen that the FSR sensor gives a near transient resistance in the range of 80–100 kPa with the highest sensitivity; however, once it is over the range, its resistance hardly changed. Such a narrow measurement range cannot meet the demand of the force contact state sensing for the high-loading legged robot. As a comparison, our piezoresistive sensor shows a more stable and wider response curve (20 Pa–800 kPa), as well as the calculated sensitivity of 0.66 kPa^−1^ (0–100 kPa).

### 2.2. Working Principle

**Figure 4:**
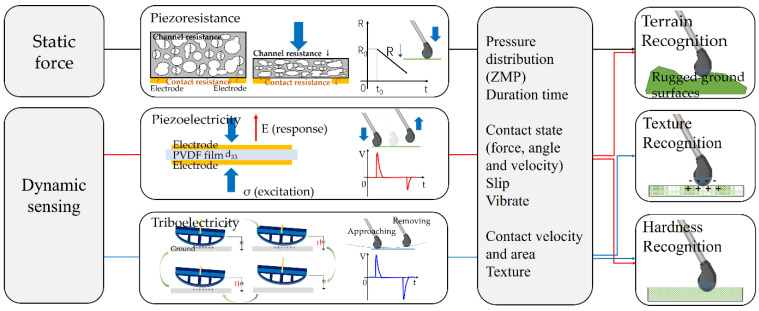
The principle of distinct signals generated from different ground contacts by the tri-modal sensor.

The piezoresistive sensor composed of porous conductive TPU/CB sponge and interdigitated electrodes serves as a static pressure detector (Figure 4). With the homogeneous hierarchically porous microstructure, there will be an increased area of contact between conductive walls under pressure loading, resulting in a decrease of both the contact resistance and channel resistance. Benefiting from the low modulus and a great many available contact surfaces, the sensing cell can be efficiently deformed under external pressure to improve the sensitivity of the device without losing the sensing range. Based on the optimized layout of cells, the ZMP of the robot can be calculated by the pressure distribution value of four discrete points, thereby realizing the stability criterion for a walking legged robot.

Meanwhile, the piezoelectric sensor is highly sensitive to high voltage outputs, even to small dynamic contact deformation/force. A representative piezoelectric sensor element has the same construction as the capacitance-based sensor, where the dielectric material (PVDF) has a negative d_33_ value [26]. However, if a load is maintained, then the sensor output decays to zero. Therefore, the piezoelectric sensor is more suitable to act as a flexible, self-powered, and lightweight dynamic force sensor to perceive the contact state between the foot and ground. Furthermore, the triboelectric sensor composed of an EVA foam friction layer and conductive cloth electrode was employed for the dynamic sensing, including contact velocity, area, and texture. As the outer surface of FMSS, when the foot contacts the ground, the charge would transfer between the surfaces since the materials possess the ability to gain or lose electrons [27]. The alternating voltage is generated when the contact–separation process occurs on the device between the triboelectric electrode and the grounded electrode [28].

The vertical packaging of identical 3D lattices guarantees that one mechanical stimulus converts into multimodal sensing signals concurrently. Hence, these three signals complement each other to form a tri-modal judgment that apprehends every walking process on a complex ground not only from the contact state but also the triggering process.

### 2.3. Experiment and Calibration

**Figure 5:**
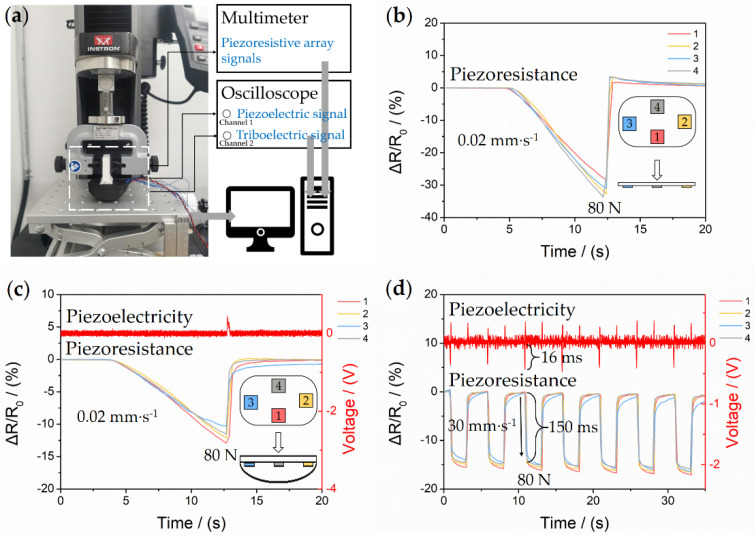
Calibration and performance results. (**a**) Experimental apparatus provides a precise and controllable load to the FMSS. Relationship between resistance responses and applied pressure of (**b**) the original piezoresistive sensors and (**c**) the packaged FMSS. Four different colors represent the load distribution in different locations. (**d**) Piezoelectric and piezoresistive signals generated by a dynamic cycle testing at a load speed of 30 mm/s and a cycle frequency of 0.2 Hz.

A static calibration system was employed to describe the basic performances of the piezoresistive array, as shown in Figure 5a, including an Instron 5943 electronic universal testing machine, a Keithley DMM6500 digital multimeter with a plug-in scanner card to provide data acquisition (DAQ) of four channels, and computer with the visual pressure display software. Under a vertical loading of 80 N (the weight of a normal quadruped robot divided by four), four piezoresistive cells with approximate resistance response curve fitting can reach the sensitivity of 1.05%/N, as shown in Figure 5b. The sensitivity of packaged FMSS, 0.15%/N, is lower than its original data because of the precompression process during assembling. However, the performances are adequate for most of the legged robots with a heavy weight (10–40 kg) and present excellent linearity in the total range of testing (Figure 5c). In order to prove the robustness and resilience of the assembled sensor, a dynamic testing cycle was implemented. An 80 N force with a loading speed of 30 mm/s was circularly applied to the sensor-mounted foot to mimic the walking locomotion (Figure 5d). The result demonstrates the synchronicity between piezoresistance and piezoelectricity. The observable piezoelectricity signal appears only at on and off moments of the dynamic stimulus, and it is difficult to be induced by a mild static stimulus. Response times of piezoelectricity and piezoresistance are 16 ms and 150 ms, respectively, and the durability of piezoresistance has been demonstrated by our earlier studies [25].

## 3. Results

### 3.1. Terrain Recognition

**Figure 6:**
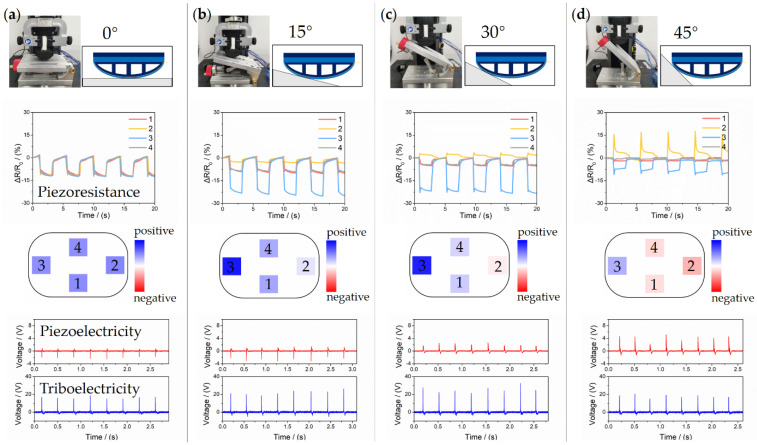
Analysis of specific response behaviors to terrain recognition. Tri-signal generated from hitting different inclinations under vertical force, including: (**a**) 0, (**b**) 15, (**c**) 30, and (**d**) 45°.

To test the FMSS mounted on the robot foot, different experiments were carried out by mimicking the kick process of the robot. The sloping testing platform placed under the electronic universal testing machine was adjusted to 0, 15, 30, and 45° to represent typical terrains. The varying distribution of pressure caused by the terrain change was measured by the piezoresistive array. In each condition, FMSS’s electronic responses of tri-modal and visual pressure gradation distributions (based on the pressure resistance relation curve of Figure 5c) were recorded in Figure 6. It is well known that a force sensor array can detect a two-dimensional distributed load, and in turn, can calculate the ZMP and shear force. As seen, the pressure of the proximal lattice experiences a higher concentration gradient (positive) compared to the distal lattices when the angle changes from 0 to 30°. As the inclination further increases, the pressure gradually turned into the pushing force (negative) because of the arcing configuration of FMSS. This changing process of force vector (from “press” to “push”) is also recorded by the variable polarity and amplitude of the piezoelectric signals. Although the amplitude-angle correspondence is nonlinear, through methods of machine learning and deep learning, the result can help improve the robustness of the force angle and distribution calculation from the piezoresistive array. However, triboelectricity retains the same characteristic waveform; the slight change of intensity is due to the different contact areas between FMSS and the testing platform. Therefore, by using the simultaneous extraction of piezoresistive and piezoelectric signals, the hybrid tactile sensor can accurately identify contact state and pressure distribution caused by rugged ground surfaces and motion patterns and has great potential in intelligent real-time feedback control of robotic locomotion.

### 3.2. Texture Recognition

**Figure 7:**
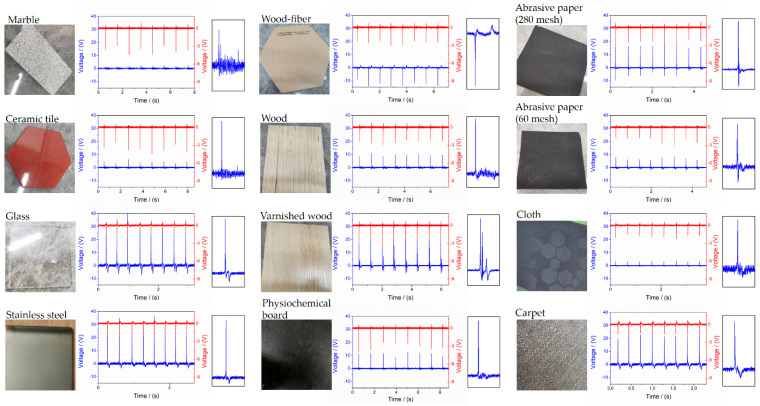
Analysis of specific response behavior to texture recognition. The double signal is generated from hitting different textures under vertical force.

Apart from the vision-based surface texture perception [29], which is easily disturbed by illumination conditions and oscillation, and force sensing resistor array (FSRA) with k-nearest neighbor (kNN) algorithm [30], which can only use on relatively flat ground with fully and evenly contact, FMSS also recognizes materials’ features from the wave patterns when the triboelectricity changes caused by simple contact. To characterize the surface texture, descriptions of 12 kinds of familiar ground were compared firstly (Figure 7). The different texture surfaces were vertically hit by FMSS to imitate the action of kicking. The synchronously collected piezoelectric signals were used as a reference to prove the similar dynamic force. Focus on the triboelectricity, gain (or loss) between EVA, and different surface nature can be reflected by the voltage amplitude of approaching and removing. It indicates that textures with the same attributes tend to gather in a region. In addition, the surface roughness of the texture can affect the intensity of the voltage amplitude without changing the waveform. For example, the 280-mesh abrasive paper displayed a stronger signal than 60-mesh one. In the following work, we will try to generalize the texture features by a model trained on much more data to achieve autonomous recognition.

### 3.3. Hardness Recognition

**Figure 8:**
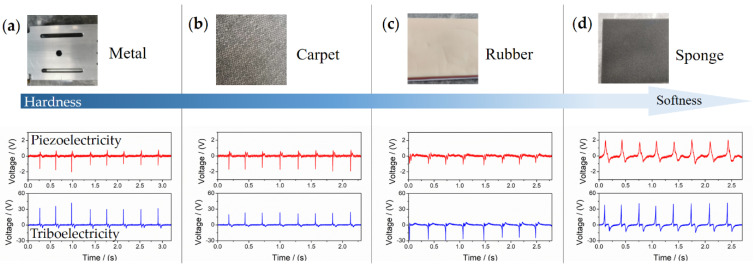
Analysis of specific response behaviors to hardness recognition. The double signal is generated from hitting different objects under vertical force, including: (**a**) metal, (**b**) carpet, (**c**) rubber, and (**d**) sponge.

In order to recognize the hardness of an object, a squeezing process by kicking is necessary. Note that this is only possible through active perception since a static reading of tactile information is not sufficient for the hardness. Here, the dynamics of the restored sensation could help discriminate specific object characteristics. Fortunately, FMSS integrated the double-modal dynamic sensing of two different action mechanisms. For testing the sensor, we recorded the vertical hitting data from four types of objects with similar thickness, consisting of a metal platform, a carpet, a rubber block, and a sponge block, as shown in Figure 8. Contact time and waveform can reflect the hardness. Long contact time means relatively low rebound speed. For example, the highest voltage magnitude and the biggest bandwidth of piezoelectricity appeared on the deformable sponge sample, and the obvious negative voltage signals were recorded on the harder objects. Furthermore, the resolution capability of triboelectricity mainly derives from the differentiation of the triboelectric series of materials [31], texture, contact velocity, and area. For example, as the EVA is triboelectrically positive compared with the rubber, the rubber more readily obtains the electrons from the contact interface of EVA, thus a minus triboelectric voltage. Therefore, the synergy information can efficiently improve the overall recognition accuracy of ground hardness and skidding situations (dynamic contact time and vibration).

## 4. Discussion

### 4.1. Experiment on Quadruped Robot Walking

**Figure 9:**
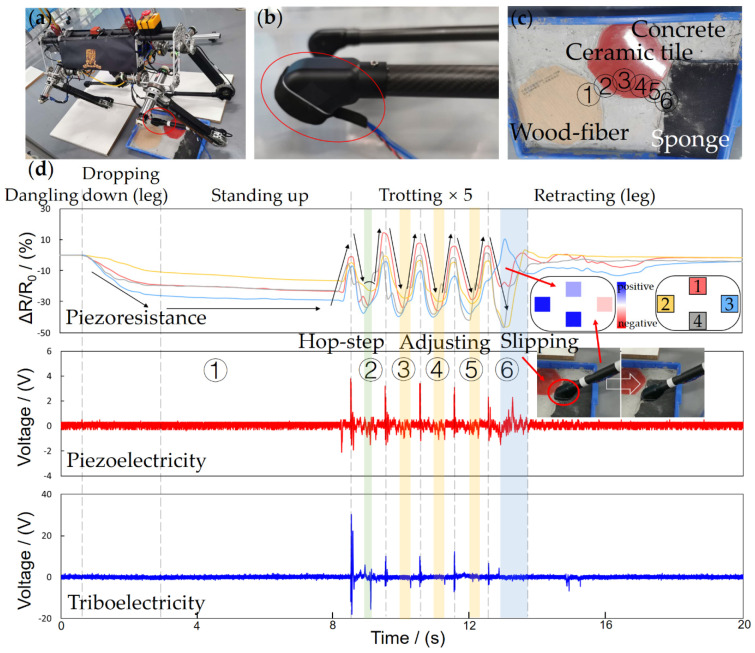
The experiment of FMSS mounted on the foot of (**a**,**b**) a large size quadruped robot Pegasus without changing the original quadruped foot and leg design. (**c**) The floor pad design with different terrains and step points follows the order from 1 to 6. (**d**) FMSS signals in the whole process, which are separated into five states according to the robot’s movements.

**Figure 10:**
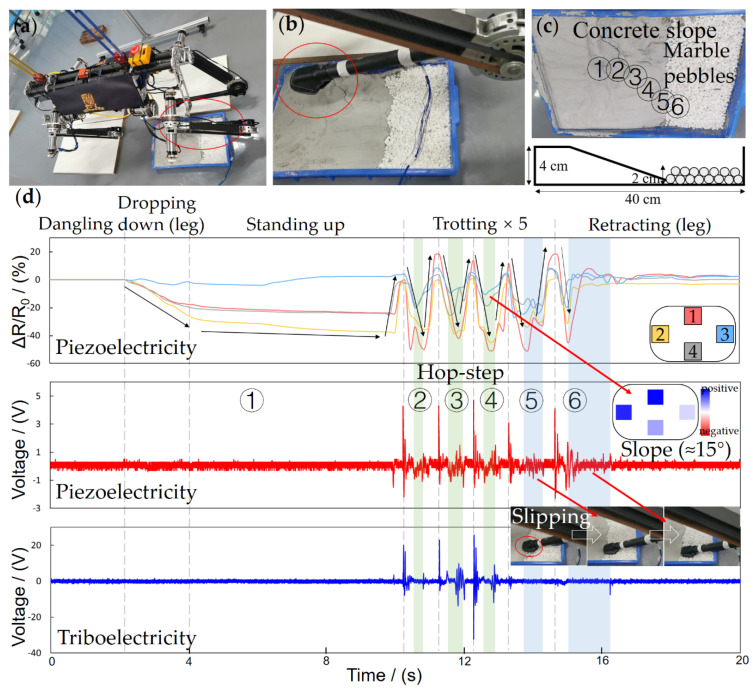
The experiment of FMSS mounted on the foot of (**a**,**b**) a large size quadruped robot Pegasus without changing the original quadruped foot and leg design. (**c**) The floor pad design with an angle and step points follows the order from 1 to 6. (**d**) FMSS signals in the whole process, which are separated into five states according to the robot’s movements.

To validate the practical performance of the FMSS, we executed tests on a large-size quadruped robot called Pegasus (about 75 mm in height and weighs 31.5 kg, Figure 9a), which possesses the ability of agile and highly dynamic locomotion, such as walking and trotting. The FMSS was mounted on one of the Pegasus’ feet without changing the leg and foot constructions, as Figure 3b shows, and the appearance shows in Figure 9b. FMSS was then tested on two different complex floor pads, respectively. The first floor is a multi-texture surface, combining concrete, ceramic tile, wood-fiber, and sponge ground (Figure 9c), and the second floor consists of coarse concrete with a 15° slope and marble pebbles (Figure 10c). Pegasus used a trotting gait trampling on the test floor pads to simulate the process of traversing complex terrain, and the FMSS raw data result was read simultaneously.

GRF distribution and terrain information was reflected in the piezoresistive array signals. Standing-up, trotting, and retracting phases corresponded to the increasing, the cycle repeating, and decreasing of relative resistance change, respectively, as seen in Figure 9d and Figure 10d. When standing separately on the horizontal floor pad one and 15° floor pad two, because of the different angles between legs and floor, sensors 2 and 3 show the sole force distribution (in the *x*-axis direction) difference. During trotting, the array readings cannot keep symmetrical and consistent. The phase indicates the contact order in different positions of the sole, and the amplitude reflects the uneven terrain. The force vector can then be used for ZMP calculation and friction cone estimation [32,33].

Many robots set a piezoresistive sensor reading threshold for contact state judgment. When Pegasus trots at a relatively high frequency, about 1.25 Hz, the swing and stance phases are not like the ideal piezoresistive square wave shape in Figure 5d; instead, they are similar to a triangle wave due to the fast loading and unloading cycle. A large threshold setting will cause a large latency and error in the judgment of contact; meanwhile, a small threshold would lead to misjudgment because of the noise. However, the piezoelectric sensor still keeps high sensitivity to dynamic contact changes and low noise, distinguishing gait state with a switch signal. In dropping-down and standing-up states, the slow and gentle contact will not cause a piezoelectric peak, but in these states, the piezoresistive sensors with a threshold method are good enough for the contact judgment. In the trotting state, as seen in Figure 9d and Figure 10d, every quick retreat is recorded as a pulse with a big negative amplitude, and every quick contact is recorded as a pulse with both big positive and negative amplitude. Even the unstable trembles are recorded in pulses.

For robots only equipped with FSR, when a slip happens, the sudden drop of contact force cannot reach the threshold instantaneously, making the robot leg stay in a swing phase, easily resulting in stability failure and slip recovery algorithm infeasibility. Thus, a slip test is designed in the experiment. During the slip in the 6th step, the piezoresistive array’s phases are out of sync, which indicates the contact condition is poor, and the piezoelectric signals oscillate violently, which indicates there is a high dynamic condition. The abnormal signal combination provides a robust judgment of slip. During the 5th step on floor pad one, from the concrete to the sponge surface, slippage happened due to the sudden texture and deformation change. Multimodal FMSS clearly tracked the slip state with a delay of the signal peak in the rear piezoresistive sensor 3 and an abnormal oscillating piezoelectric signal (Figure 9d).

The piezoresistive signal comparison also illustrated the terrain condition. The pad slope of about 15° can be distinguished from the differences between piezoresistive array readings of sensor 2 and sensor 3 in general. The coarse and uneven terrain surface was loyally recorded in small bumps as well. On the other hand, the composite signal of force distribution superposition indicates shear forces on the contact surface. The shear force allows a robot to directly and sensitively monitor changing terrain and incipient slips compared to the velocity and acceleration-based slip detector. Furthermore, the texture can be recognized to a certain extent through the triboelectric unique signal waveforms when FMSS steps on it.

In all, the rich multimodal signals of FMSS enable legged robots to extract rich information about the unstructured environment and help achieve high dynamic and stable movement.

### 4.2. Experiment on Human Walking

**Figure 11:**
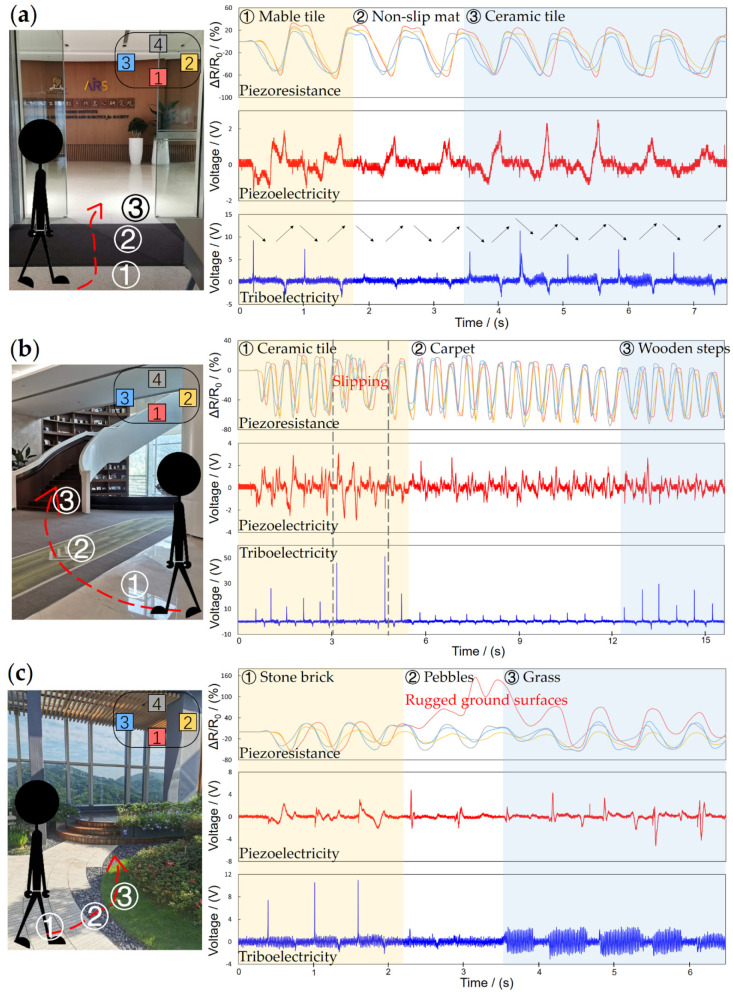
The experiment of FMSS mounted on the bottom of the tester’s foot. The traverse paths and signal result in a typical (**a**) hall, (**b**) office, and (**c**) garden scenario.

Learning locomotion from animals and human beings is also a hot research direction. FMSS can be used as an important supplement in analyzing and imitating human walking. The FMSS was mounted on one of the human tester’s shoe soles to record the tactile feedback of human locomotion. Then the human tester equipped with FMSS on the sole walked through three scenarios that included some typical kinds of terrains, as shown in Figure 11. Different from robots, the piezoresistive array readings vary in every step cycle, and four sensors in the array show less synchronization than a robot, which means humans adapt their gait and contact location order constantly according to the slightest change. The piezoelectric signal pulses are gentler compared to the experiment on the quadruped robot but still clear enough for the state switch judgment, which pictures a soft-landing process different from a simple mass-spring-damper model. The textures can preliminary be distinguished by the triboelectric signal wave patterns, which have shown a strong feature in the wave pattern (for example, in Figure 11c, stone brick, pebbles, and grass). FMSS proved its effectiveness and adaptability in a more complex application scenario. What is learned from human locomotion through FMSS may provide new ideas and control strategies for agile robot maneuvers [34].

## 5. Conclusions

Tactile information is important to achieve agile maneuvers for legged robots. The effectiveness and adaptability are the key features for a sensor to be applied to a highly dynamic robot. From the experiments, FMSS provided high dynamic reliability, consistent and direct available tactile signals without changing the robot’s mechanical design. Different signals from FMSS provided key features, such as pressure distribution, contact state, and texture during foot–ground interaction, while multimodal signals pictured some higher dimensional useful tactile features, such as shear force, hardness, slippage. We expect that this study can be very handy in ontology status estimation and environment detection. Moreover, with the development of reinforcement learning and sensor fusion in robot control, abundant ground information from multimodal sensors, such as FMSS, has the potential for a wide range of applications in legged robots.

## Data Availability

The data presented in this study are available on request from the corresponding author.
